# Natural malaria infection in anophelines vectors and their incrimination in local malaria transmission in Darién, Panama

**DOI:** 10.1371/journal.pone.0250059

**Published:** 2021-05-03

**Authors:** Rolando Torres-Cosme, Chystrie Rigg, Ana María Santamaría, Vanessa Vásquez, Carlos Victoria, José Luis Ramirez, José E. Calzada, Lorenzo Cáceres Carrera

**Affiliations:** 1 Departamento de Investigación en Entomología Médica, Instituto Conmemorativo Gorgas de Estudios de la Salud (ICGES), Panam, Repblica de Panam; 2 Departamento de Investigación en Parasitología, Instituto Conmemorativo Gorgas de Estudios de la Salud (ICGES), Panam, Repblica de Panam; 3 Departamento de Control de Vectores, Ministerio de Salud (MINSA), Panam, Repblica de Panam; 4 Crop Bioprotection Research Unit, National Center for Agricultural Utilization Research, Agricultural Research Service, United States Department of Agriculture, Peoria, Illinois, United States of America; Instituto Nacional de Salud Pública, MEXICO

## Abstract

**Background:**

More than 85% of the malaria cases in Panama occur in poor, rural and indigenous regions like Darien Province. Vector diversity, infection rate and spatial distribution are important entomological parameters of malaria transmission dynamics. Their understanding is crucial for the development of effective disease control strategies. The objective of this study was to determine the composition of *Anopheles* species, their natural infection rate and their geographic distribution to better understand the malaria transmission dynamics in Darién, Panama.

**Methods:**

Anophelines mosquitoes were captured during the rainy and dry season of 2016. We selected five communities where adult anophelines were collected using CDC light-traps, and through protective human-baited traps. Detection of natural infection and *Plasmodium* genotype were detected via nested PCR through the amplification of ssrRNA and the circumsporozoite protein gene (csp), respectively.

**Results:**

A total of 1,063 mosquitoes were collected mosquitoes were collected for the detection of natural infection with *Plasmodium spp*. Nine Anophelines species were identified, with the predominant species being: *An*. *(Nys*.*) darlingi* (45.0%) and *An*. *(Nys*.*) albimanus* (42.6%). Natural infection in *An*. *(Nys*.*) albimanus* with *P*. *vivax* was detected in one mosquito pool from the community Pueblo Tortuga (0.6%), three from Marraganti (1.7%), two from Bajo Chiquito (1.1%) and three pools from Alto Playona 3 (1.7%). For *An*. *(Nys*.*) darlingi* mosquitoes, we detected seven positive pools from the community Bajo Chiquito (4.0%), two pools from Marraganti (1.1%) and two pools from Alto Playona (1.1%). The *P*. *vivax* allelic variant VK210 was detected in infected mosquitoes.

**Conclusion:**

The results from this study provide new information on the transmission dynamics associated with anophelines vectors in the Darién region. This is the first report of natural *P*. *vivax* infection in *An*. *(Nys*.*) darlingi* and its incrimination as a potential malaria vector in this region of Panama. Additional studies are necessary to expand our knowledge and determine crucial parameters in malaria transmission in Darién, which in turn will aid the National Malaria Program in attaining an adequate malaria control strategy towards malaria elimination.

## Introduction

Malaria is one of the most important public health problems; in 2018 it caused more than 228 million cases and 405,000 deaths according to the World Health Organization (WHO) [1]. This disease affects the health and work capacity of large number of people due to its vast geographic distribution, morbidity, mortality and socio-economic impact [2]. Malaria transmission currently exists in 21 countries and territories of the American continent. In these areas, 132 million people are at risk of infection, while 21 million people live in areas of high transmission risk. According to Pan American Health Organization (PAHO), since 2015 the Americas have experienced an increase in the number of cases mainly due to a rise in the incidence in Venezuela, and to a higher transmission rate in endemic areas from countries like Brazil, Colombia, Guyana, Nicaragua and Panama. In addition, there has been recent outbreaks in countries that have been advancing towards malaria elimination (Costa Rica, Dominican Republic and Ecuador). In comparison, Paraguay and Argentina have received certification as malaria-free countries in July 2018 and May 2019, respectively. Also, is worth mentioning the scenario in El Salvador and Belize, who have presented no autochthonous cases since 2017 and 2019, respectively [3].

Despite the regional efforts, malaria in Panama continues to represent an important public health problem [4], with active transmission limited to 10 municipalities (12.5% of the total Panamanian municipalities), and with the poorest rural regions (mostly indigenous), being the most affected population. This is in part due to the geographical isolation and the lack of access to public health services, poverty and illiteracy. Indigenous regions, which occupy 22% of the national territory and only 12% of the total population, register more than 85% of the total malaria diagnosed cases [5]. The variations that occur in these regions determine the main changes affecting malaria epidemiology in Panama.

Generally, the circulation of the malaria parasites follows the spatial distribution of their anophelines vectors [6]. The genus *Anopheles* is found throughout the world and the majority of the 465 known species are not malaria vectors. Approximately, 41 species of the genus Anopheles are associated with *Plasmodium* transmission to humans [7]. In Panama, *An*. *(Nys*.*) albimanus* is of great entomological and epidemiological importance given that its predominance and role as primary malaria vector along the coastal areas of the Caribe and the Pacific. *Anopheles (Ano*.*) punctimacula*, is known as an important secondary vector. Nevertheless, there are other anophelines species that could be implicated in malaria transmission in the different endemic regions of Panama. For instance, *An*. *(Nys*.*) darlingi*, has recently been described in specific regions of Panama [8]. However, it is the primary malaria vector of much of Latin America and is known to readily colonize habitats with diverse ecological characteristics. Furthermore, depending on the environment, this mosquito species displays a range of behaviors: anthropophily, opportunism, endo-exophagy, and endo-exophily. It also frequently colonizes anthropogenic sites and is susceptible to *P*. *vivax* and *P*. *falciparum* [9, 10].

The determination of natural *Plasmodium* infection in anophelines mosquitoes is an important step to discern the main malaria vector species that exists in a region [11]. In Panama, experimental infections with human blood infected with circulating gametocytes, have found that seven *Anopheles* species are susceptible to infection with either *P*. *vivax* or *P*. *falciparum*. Nevertheless, only four of these species have been found to be naturally infected in the wild with *Plasmodium spp*. oocysts and/or sporozoites [12–16]. Currently there are 26 *Anophele*s species described in Panama [17–19]. The species *An*. *(Ano*.*) malefactor*, was also included by Wilkerson and Strickman [19] following a systematic revision differentiating this species from its synonym *An*. *(Ano*.*) punctimacula s*.*l*. [18]. Additionally, a recent report described the presence of *An*. *(Nys*.*) darlingi* in two communities in Darién province located at the eastern end of the country bordering with Colombia [20].

Social, environmental, economic, demographic and climatic changes can significantly influence the distribution of anophelines in endemic areas [21–23]. Although some species have been incriminated as local or regional vectors in neighboring countries [24], their importance as malaria vectors in Panama has yet to be determined. Additional entomological studies evaluating the vectorial capacity of other anophelines species are needed to determine their epidemiological importance in each of the Panamanian endemic regions. Equally important is the frequent monitoring of entomological parameters such as abundance, composition and natural infection with *Plasmodium*. Information gathered in these areas will contribute to the improvement of regional and local vector control strategies that can lead to malaria elimination in Panama. In this regard the objectives of this entomological study were to determine the species composition, spatial distribution, detection of natural *Plasmodium* infection and genotype, and their association with dominant mosquito species in Darién. This study provides new information on malaria transmission dynamics and has great public health significance allowing a more adequate selection of vector control interventions in this endemic region of Panama.

## Material and methods

### Description of the study area

The collection of adult *Anopheles* mosquitoes was conducted in the indigenous comarcas Emberá-Wounaan and Wargandi, located in Darién. The term comarca refers to an administrative region within Panama and it is assigned to a given indigenous population. The eastern side of Panama, where Darién is located, has seen 61.5% of national malaria cases in the last years [5]. The climate of this region is tropical, with high humidity like the rest of Panama, and it is influenced by the intertropical convergence zone [25]. Darién has two well defined seasons with moderate high temperature and humidity: the dry season (January—April) and the rainy season (May—December). The average annual precipitation ranges from 1,700 to 2,000 mm, with May and November being the rainiest months. The temperature does not present significant variations, ranging from 25.6 to 27.1°C. This region registers a relative humidity of 84% and it ranges from 75% (March, dry season) to 93% (November, rainy season) [26]. According to the system of biosphere classifications by Holdridge, Darién belongs to a tropical rainforest [27].

### Study design and site selection

**Fig 1:**
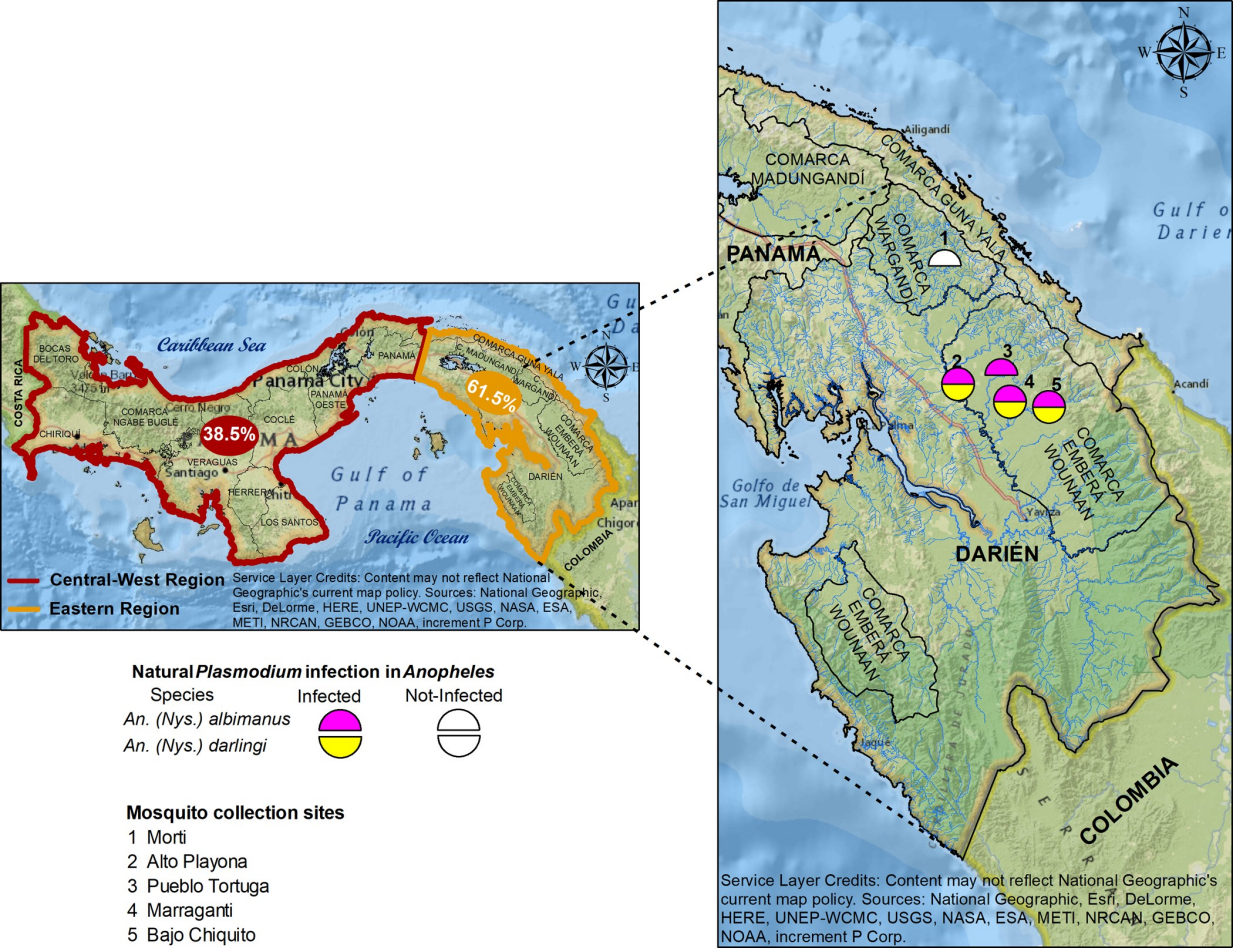
Geographical location of the studied communities in the Darién region, Panama. The Map was created using the software ArcGIS Desktop 10.7.1 and ArcGIS Online.

Site selection was conducted in conjunction with technical personnel from the National Malaria Program (NMP) of the Ministry of Health (known as MINSA in Spanish). The selected sites were indigenous communities that presented active malaria transmission: Marraganti, Bajo Chiquito, Pueblo Tortuga and Alto Playona, located in the comarca Embera-Wounaan, and Morti in the indigenous comarca Guna of Wargandi (Fig 1 and Table 1). All the communities were characterized by being located near a river that are used as the main communication means with other nearby communities, surrounded by an ecology of lowlands, creeping vegetation and secondary forest. Most households in this area presented wooden walls, tin roofs and a few were protected from insect entrance. Selection of communities was based on the following criteria: (1) its entomological importance with regards to vectors, (2) its epidemiological importance according to the transmission rate and (3) accessibility and safety to visit the community, as the presence of irregular armed groups and drug traffickers is frequent in certain areas from Darién.

**Table 1:** Epidemiological information from the studied communities in Darién, 2016.

Comarca	Community	Coordinates	Altitude	Households	Population	Malaria Cases
Embera-Wounaan	Marraganti	8°27’56.15" N	50 m	95	390	8
		77°41’54.77" W				
	Bajo Chiquito	8°27’22.17" N	40 m	48	180	4
		7°40’45.84" W				
	Pueblo Tortuga	8°32’2.32" N	71 m	42	190	2
		77°42’44.53" W				
	Alto Playona	8°32’20.02" N	49 m	67	215	2
		77°52’56.55" W				
Wargandi	Morti	9° 10’13.29" N	11 m	80	395	20
		78°47’58.72" W				
Total				479	1,755	36

#### Comarca: The term comarca refers to an administrative region within Panama and it is assigned to a given indigenous population

We designed a cross-sectional study to identify the species of *Anopheles* vectors present in the selected communities and to detect natural *Plasmodium* infection and genotype in these vectors. Mosquitoes were collected via two methods in each of the selected communities where malaria cases were registered: protective human-baited capture (PHBC), which consists of exposing only one limb and capturing the mosquito upon landing before the bite begins [24]; and CDC light traps (LTs). Each locality represented a sampling site, and for each selected location mosquito samplings were conducted for nine months; twice during the dry season (March and April) and twice during the rainy season (May to November) of 2016. The NMP provided the following epidemiological data: geographical location of the malaria cases, clinical characteristics of the disease, date of the first cases, affected and exposed population, socioeconomic information, basic sanitation services, health service characteristics, demographic information and prevention and control interventions carried out in the region. This information was important to select the study sites, based on the communities that registered higher malaria transmissions in recent years.

### Collection of adult mosquitoes

To collect adult mosquitoes, four houses that presented malaria or were near to a mosquito breeding sites were selected. Mosquito collection in each of the sites were conducted at different dates but within the same dry and rainy season. Mosquito collections with PHBC were carried out in the five selected communities during four consecutive nights in the intra and peridomicile areas simultaneously once a month, for nine months including the dry (March and April) and rainy seasons (May to November) of 2016. To determine the human biting rate per night (HBRs), the PHBC consisted in simultaneously having one-person intradomicile and another in the peridomicile per night in the four households within each locality. Mosquito collections were conducted during the 18:00 and 22:00 hours, given that previous reports have indicated that this is the period of greater mosquito biting activity [28, 29]. Collections were made during the first 50 min of every hour. During this period, HBRs were calculated, such as the biting/human/night (BHN) rate during the period from 18:00 p.m. to 22:00 p.m. [30]. The biting rate was obtained from the total number of mosquitoes captured divided by the number of collectors and the total hours of captures [31]. Mosquito collections were conducted by experienced technical personnel from the NMP and the Medical Entomology Department from the Gorgas Memorial Institute for Health Studies, trained in the capture of malaria mosquito vectors. To reduce bias during collections, personnel were rotated at specific periods every hour. The technical biosafety recommendations from WHO [32] were followed to minimize the risk of malaria infection in the field team.

Mosquito collections using LTs were also conducted during four consecutive nights simultaneously to the PHBC collections. The (LTs) were located at the height of 1.5 m above the ground during the hours of 18:00 and 6:00 [33, 34]. Neither octanol nor carbon dioxide (CO_2_) were used as mosquito attractant. Traps were distributed near houses (different from the homes where PHBC collections were made) and mosquito breeding sites (extradomicile) to a distance about 25 m. Collections with PHBC were used to measure the intensity of the anophelines bite, while the LTs were used to improve the opportunity to collect a greater variety of species of [35, 36]. Additional parameters such as temperature and relative humidity were recorded during the periods of mosquito collection using a data logger (EXTECH instruments® RHT10, No. 11092261). Rainfall data were obtained from a meteorological station in the locality of Yaviza, near the collection sites.

### Taxonomic determination

All biological material collected was placed in previously labeled containers and preserved in liquid nitrogen (Thermo Scientific™). Each container had information such as locality, type of collection (intra, peridomicile and LTs) and date. Samples were sent to the Department of Medical Entomology at the Gorgas Institute for its identification. Mosquito identification to the species level was conducted using taxonomical keys for adult mosquitoes [19, 37] and the Reference Collection from the Gorgas Memorial Institute for Health Studies.

To confirm the identity of *Anopheles* species in *Plasmodium* positive pools, the mitochondrial COI gene was analyzed using primers UEA3 (5-′TATAGCATTCCCACGAATAAATAA-3′) and UEA10 (5-′TCCAATGCACTAATCTGCCATATTA-3′) and PCR conditions as previously described [20, 38, 39]. The PCR-amplified products from these samples were excised from agarose gel and purified using the Qiaquick gel extraction Kit (Qiagen, CA, USA) following the manufacturer’s instructions. DNA sequencing of both strands was carried out using the same primers with an ABI Prism 3500 XL130 sequencer (Applied Byosistems, Foster City, CA, USA). Resulting sequences were edited using Sequencher software. The DNA sequences were compared with Anopheles COI sequences available in the GenBank by performing a BLAST search from the National Center for Biotechnology Information Database (http://www.ncbi.nlm.nih.gov/BLAST/).

### Detection of *Plasmodium spp*. genotype and natural infection in *Anopheles spp*. mosquitoes

Adult female mosquitoes were grouped per species in anophelines pools. Each pool was made of two to five female mosquitoes of the same species, according to the date and site of collection, and type of capture (PHBC or LTs). It has been described that it is possible to detect infectivity in mixtures of mosquitoes; thus, complete mosquitoes of the same species were processed [40–44]. Each pool was placed in an Eppendorf tube, where they were first macerated in a solution of 180 μl of 1X PBS. Next, DNA extraction was performed following the protocol from Qiagen DNeasy Blood & Tissue (Qiagen, USA). The isolated DNA was stored at -20°C until further use.

We used a nested PCR that amplifies the small subunit ribosomal RNA (ssrRNA) genes to confirm the presence of natural infection of *Plasmodium spp*. in pools of *Anopheles spp*. mosquitoes, following a slightly modified methodology proposed by Snounou et al. [45]. Two genus-specific primers rPLU5 (5′-CCT GTT GTT GCC TTA AAC TTC-3′) and rPLU6 (5′-TTA AAA TTG TTG CAG TTA AAA CG-3′), are used for the first cycle of amplification. An aliquot of the product obtained is used for a second amplification cycle, in which each parasite species is detected separately using species specific primers. In the second PCR reaction only specific primers for *P*. *falciparum* (205 pb): rFAL1 (5′-TTA AAC TGG TTT GGG AAA ACC AAA TAT ATT-3′), rFAL2 (5-′ACA CAA TGA ACT CAA TCA TGA CTA CCC GTC-3′) and *P*. *vivax* (120 pb) rV1V1 (5′-CGC TTC TAG CTT AAT CCA CAT AAC TGA TAC-3′), rV1V2 (5′-ACT TCC AAG CCG AAG CAA AGA AAG TCC TTA-3′) were included, because the other *Plasmodium* human species (*P*. *ovale* and *P*. *malarie)* have not been reported in Panama in more the four decades. All mixture and amplification conditions were as described previously [45].

The circumsporozoite protein gene (*csp*) was used as marker to genotype *Plasmodium vivax* in positive pools of natural infected *An*. *(Nys*.*) darlingi* and *An*. *(Nys*.*) albimanus*. A nested PCR amplification method was used to amplify the *csp* gene (700 to 800bp) following previously reported protocols with some minor modifications [46]. The primers were as follows: VCS-OF (ATGTAGATCTGTCCAAGGCCATAAA), VCS-OR (TAATTGAATAATGCTAGGACTAACAATATG) as primary primers and VCS-NF (GCAGAACCAAAAAATCCACGTGAAAATAAG), VCS-NR (CCAACGGTAGCTCTAACTTTATCTAGGTAT) as a nested primer. All mixture and amplification conditions were as described previously [46]. The nested PCR products were electrophoresed on a 1.5% agarose gel and then directly sequenced in both directions using an ABI Prism 3500 XL sequencer (Applied Byosistems, Foster City, CA, USA). The sequences were edited and aligned with Sequencher 4.1.4, and Phylogenetic trees were made using the Molecular Evolutionary Genetics Analysis (MEGA) 7.0.

### Data analysis

The abundance of each species of mosquito per community was expressed as a percentage, obtained from the number of each species of mosquito captured in each community among the total of the species captured in all communities. The percentage of *Plasmodium* infected *Anopheles* was assessed by the number of positive *Anopheles* specimens of a given species (np) out of the total analyzed by community (nt) by 100 [IR = (np/nt) × 100]. The natural infection rate (IR) was also estimated by the method of pooled prevalence for variable pool size and perfect tests using the pooled prevalence calculator (https://epitools.ausvet.com.au/ppvariablepoolsize). In addition, the confidence interval (CI 95%) was calculated to indicate the reliability of the estimated value. The BHN activity was registered directly from PHBC. Hourly data from all collections were grouped and the total number of bites per hour was obtained for each species by intradomiciliary and peridomiciliary behavior. Mosquito species mean density for *Anopheles* species with more than five specimens per locality, was calculated as the geometric mean of the number of mosquitoes captured per person each night.

### Ethical considerations

The methodology used to collect the mosquitoes was approved by the Technical and Institutional Ethics Committee of the NMP of the Ministry of Health (MINSA) (Note No. 25/DCV/DGS/MINSA/2016). Samples and human data were not used.

## Results

### Biting activity

The collections yielded 1,063 female adult mosquitoes, from which we were able to identify nine species (Table 2). The most predominant species were *An*. *(Nys*.*) darlingi*, 45% (n = 478) and *An*. *(Nys*.*) albimanus* 42.6% (n = 453), followed by *An*. *(An*.*) pseudopunctipennis s*.*l*. 8.2% (n = 87), *An*. *(Nys*.*) oswaldoi s*.*l*. 1.4% (n = 15), *An*. *(Ano*.*) punctimacula s*.*l*. 0.3% (n = 3) and *An*. *(Nys*.*) triannulatus s*.*l*. 0.3% (n = 3).

**Table 2:** Abundance and natural *Plasmodium vivax* infection in mosquitoes collected in Darién, Panama.

Species Mosquitos	Embera-Wounaan				Wargandi	Total Mosquitos Species	(%) Mosquitos Species
	Bajo Chiquito	Alto Playona	Marraganti	Pueblo Tortuga	Morti		
*An*. *(Nys*.*) albimanus* (PHBC)	137	24	215	64	3	443	42.6
CDC trap	3	1	6			10	
Relative abundance %	30.9	5.5	48.7	14.1	0.6		
No. pool positives	2	3	3	1			
IR % (CI)	1.1 (-0.4–2.6)	1.7 (-0.2–3.6)	1.7 (-0.2–3.6)	0.6 (-0.5–1.7)			
*An*. *(Nys*.*) darlingi* (PHBC)	385	10	73			468	45.0
CDC trap	6		4			10	
Relative abundance %	81.8	2.1	16.1				
No. pool positives	7	2	2				
IR % (CI)	4.0 (1.1–6.9)	1.1 (-0.4–2.6)	1.1 (-0.4–2.6)				
*An*. *(Ano*.*) punctimacula* s.l. (PHBC)		1			2	3	0.3
CDC trap							
Relative abundance %		33.3			66.7		
No. pool positives							
IR % (CI)							
*An*. *(Ano*.*) malefactor* (PHBC)	1					1	0.1
CDC trap							
Relative abundance %	100						
No. pool positives							
IR % (CI)							
*An*. *(Ano*.*) apicimacula* (PHBC)			1			1	0.1
CDC trap							
Relative abundance %			100				
No. pool positives							
IR % (CI)							
*An*. *(Nys*.*) strodei* (PHBC)							0.1
CDC trap	1					1	
Relative abundance %	100						
No. pool positives							
IR % (CI)							
*An*. *(Nys*.*) oswaldoi* s.l. (PHBC)	1	14				15	1.4
CDC trap							
Relative abundance %	6.7	93.3					
No. pool positives							
IR % (CI)							
*An*. *(Nys*.*) triannulatus* s.l.(PHBC)	1		1			2	0.3
CDC trap	1					1	
Relative abundance %							
No. pool positives	66.7		33.3				
IR % (CI)							
*An*. *(Ano*.*) pseudopunctipennis* s.l.(PHBC)	40	1	25		8	74	8.2
CDC trap	2		11			13	
Relative abundance %	48.3	1.1	41.4		9.2		
No. pool positives							
IR % (CI)							
*Anopheles (Nys*.*) spp*. (PHBC)	4	1	3			8	0.9
CDC trap	2					2	
Relative abundance %	60.0	10.0	30.0				
No. pool positives							
IR % (CI)							
*Anopheles (Ano*.*) spp*. (PHBC)			1			1	0.1
CDC trap							
Relative abundance %			100				
No. pool positives							
IR % (CI)							
*Anopheles spp*. (PHBC)	4		4			8	0.9
CDC trap	1		1			2	
Relative abundance %	50.0		50.0				
No. pool positives							
IR % (CI)							
Total	**589**	**52**	**345**	**64**	**13**	**1063**	**100**

#### IR: Mosquito infection rate

*Anopheles (Nys*.*) darlingi* and *An*. *(Nys*.*) albimanus* were present during the entire collection period presenting a maximum endophagic and exophagic behavior between 18:30 and 19:30 hours, and with a gradual decrease in activity along the collection period. All the species were collected with PHBC in the intradomicile and in the peridomicile in each of the communities. The species more frequently collected in the intradomicile and peridomicile respectively were with 195 and 273 for *An*. *(Nys*.*) darlingi*, followed by 188 and 255 for *An*. *(Nys*.*) albimanus*, 31 and 46 for *An*. *(Ano*.*) pseudopunctipennis s*.*l*., and one and two for *An*. *(Ano*.*) punctimacula s*.*l*. (Table 3). No significant differences were observed in the BHN bite rate in the intra and peridomicile areas in the species captured in the studied communities.

**Table 3:** Number of *Anopheles* species captured in the intradomicile and peridomicile in the communities sampled in the Darien Region, Panama.

Species Mosquito	Embera-Wounann								Wargandi		Total Mosquito
	Bajo Chiquito		Alto Playona		Marraganti		Pueblo Tortuga		Morti		
	Intra	Peri	Intra	Peri	Intra	Peri	Intra	Peri	Intra	Peri	
*An*. *(Nys*.*) darlingi*	163	222	3	7	29	44					468
*An*. *(Nys*.*) albimanus*	55	82	10	14	99	116	24	40		3	443
*An*. *(Ano*.*) pseudopunctipennis s*.*l*.	16	24		1	9	16			3	5	74
*An*. *(Nys*.*) oswaldoi s*.*l*.		1		6	8						15
*An*. *(Ano*.*) punctimacula s*.*l*.			1							2	3
*An*. *(Nys*.*) triannulatus s*.*l*.	1					1					2
*An*. *(Ano*.*) apicimacula*						1					1
*An*. *(Ano*.*) malefactor*		1									1
*Anopheles (Nys*.*) spp*.		4	1			3					8
*Anopheles (Ano*.*) spp*.						1					1
*Anopheles spp*.	1	3				4					8
**Total**	236	337	15	28	145	186	24	40	3	10	1024

**Fig 2:**
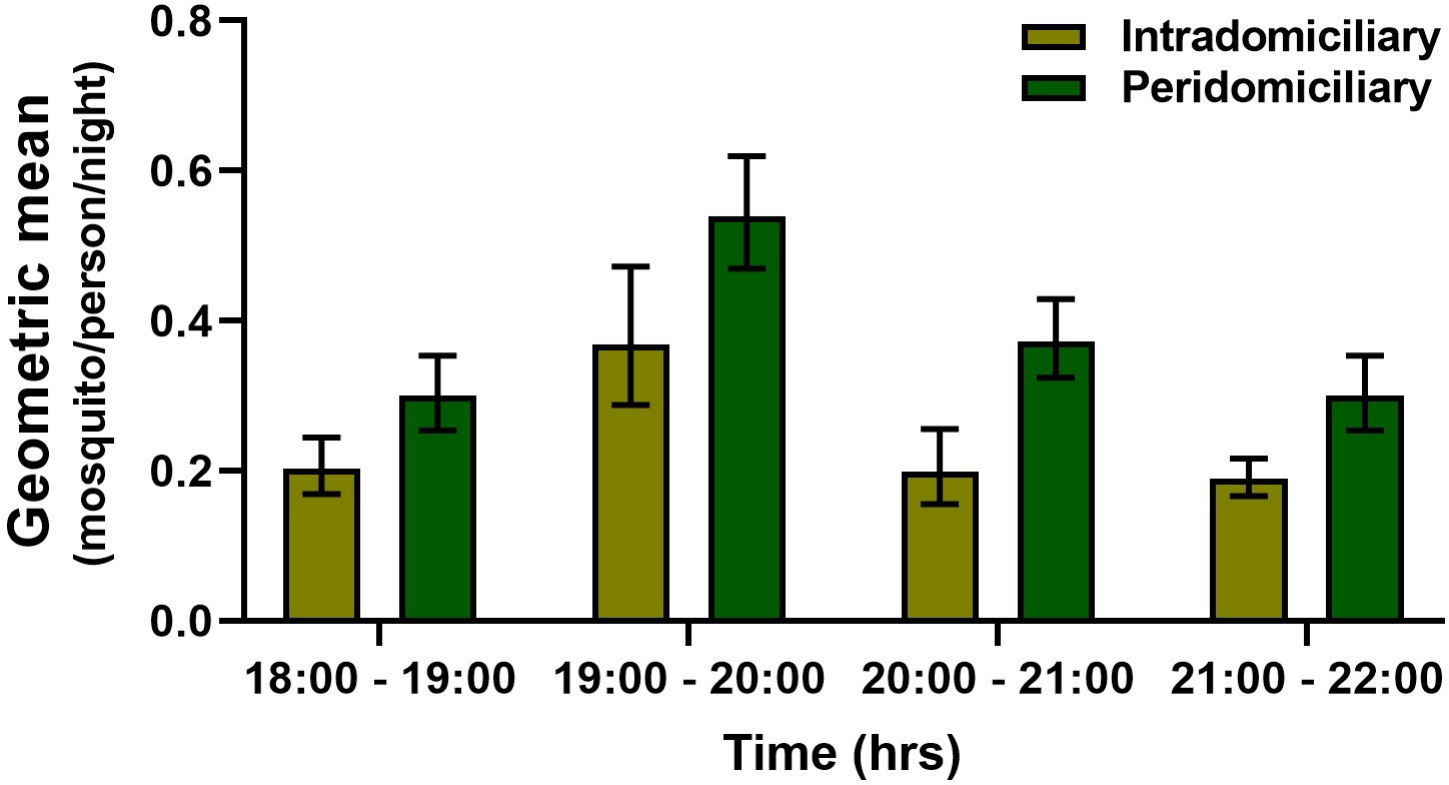
Biting activity per hour of *Anopheles (Nys*.*) darlingi* in five communities of the Darien region, between March and November 2016 (geometric mean ± 95% confidence interval).

**Fig 3:**
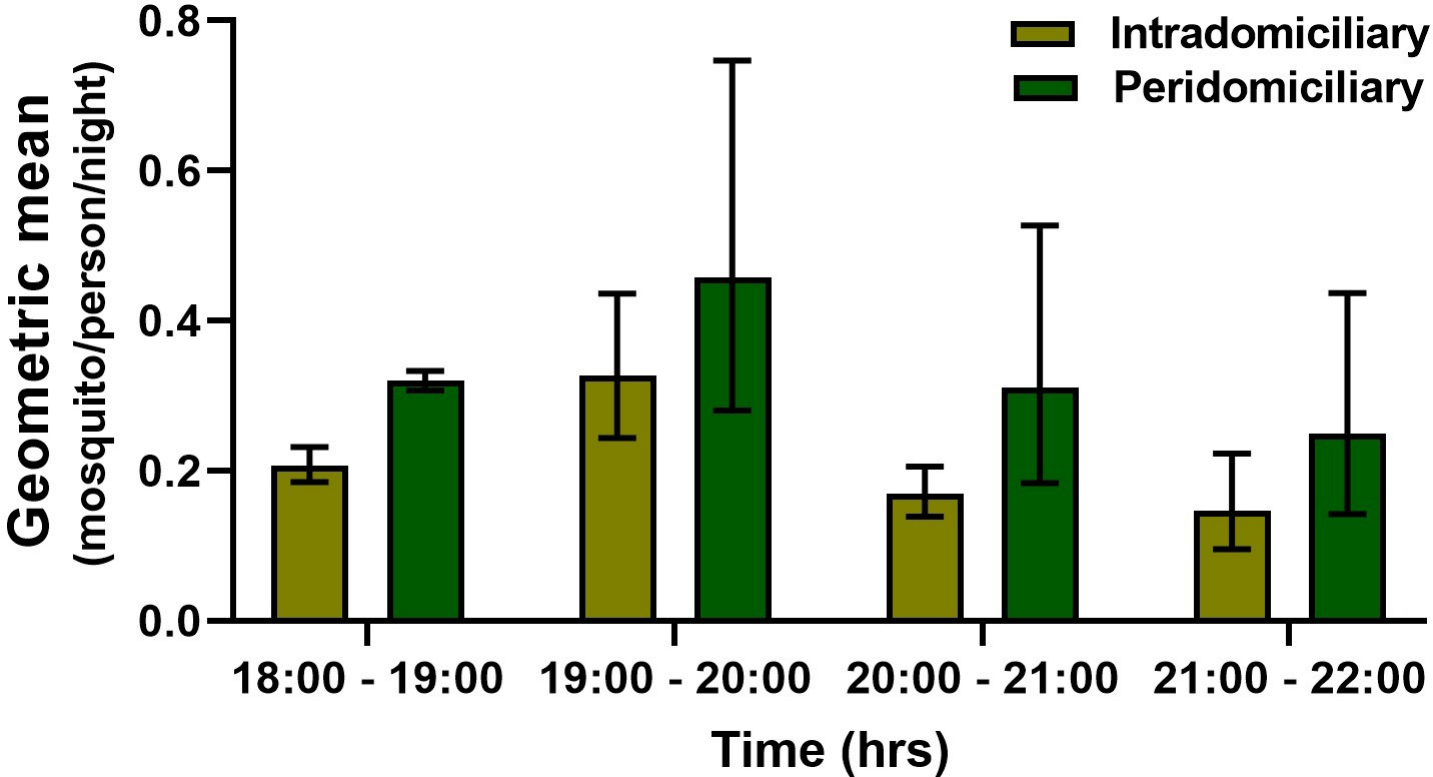
Biting activity per hour of *Anopheles (Nys*.*) albimanus* in five communities of the Darien region, between March and November 2016 (geometric mean ± 95% confidence interval).

**Fig 4:**
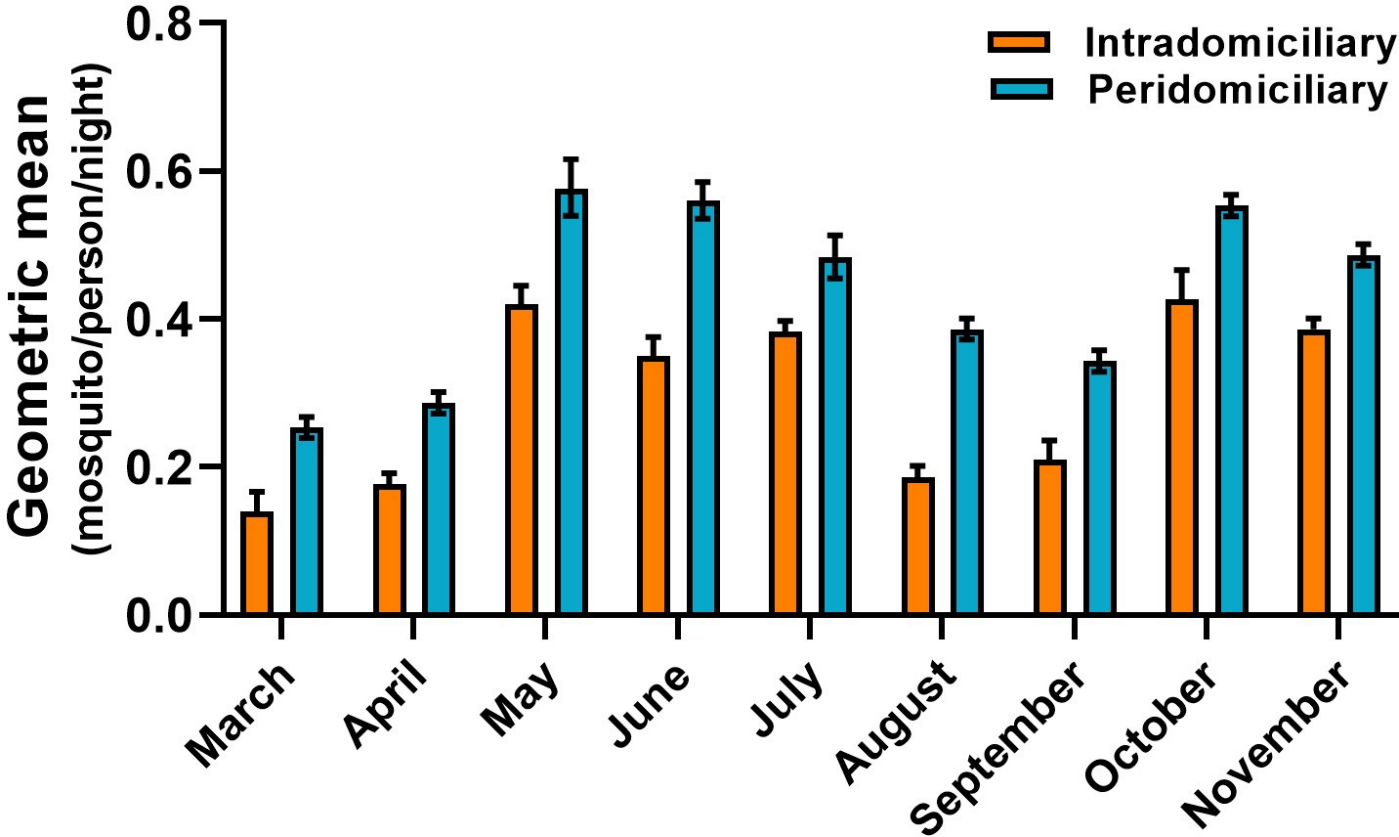
Behavior of the monthly density of *Anopheles (Nys*.*) darlingi* in five communities of the Darien region, between March and November 2016 (geometric mean ± 95% confidence interval).

**Fig 5:**
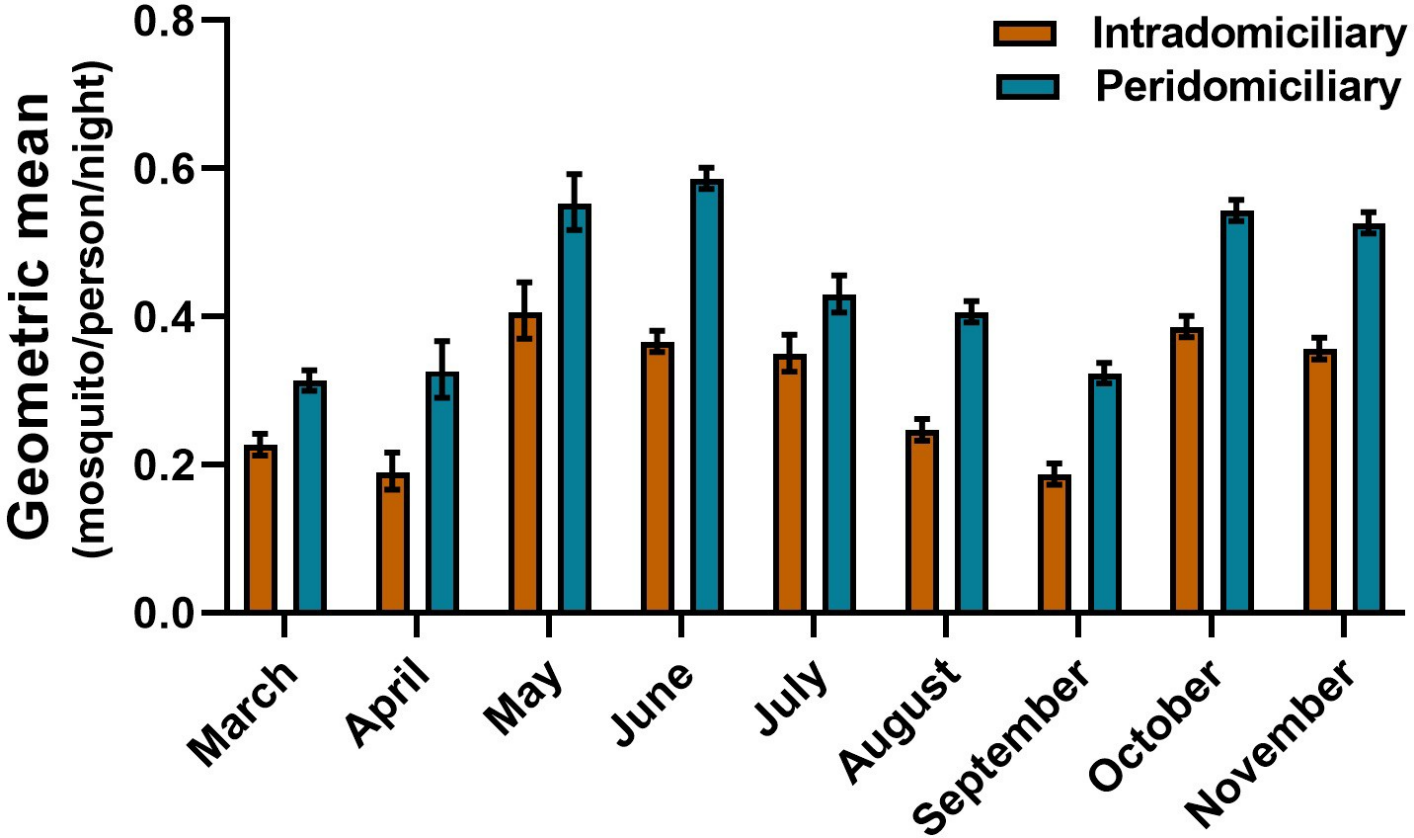
Behavior of the monthly density of *Anopheles (Nys*.*) darlingi* in five communities of the Darien region, between March and November 2016 (geometric mean ± 95% confidence interval).

The PHBC for *An*. *(Nys*.*) darlingi* and *An*. *(Nys*.*) albimanus* in the intra and peridomicile area did not show a marked difference in the BHN rates. In the five communities, an average bite rate between 0.20 to 0.39 and 0.30 to 0.55 was obtained in the intra and peri-domicile, respectively for *An*. *(Nys*.*) darlingi*, For *An*. *(Nys*.*) albimanus* it was between 0.22 to 0.40 and 0.22 to 0.56, respectively. The monthly density of *An*. *(Nys*.*) darlingi* in the intra and peridomicile was between 0.13 to 0.44 and 0.25 to 0.57 mosquito/person/night, respectively (Figs 2 and 3). For *An*. *(Nys) albimanus* it was 0.22 to 0.42 and 0.31 to 0.59 mosquito/person/night, respectively (Figs 4 and 5). Observations made in the nine months of this study, allowed to establish that *An*. *(Nys*.*) darlingi* and *An*. *(Nys*.*) albimanus* were present throughout the year with endophagic and exophagic behavior, given that they were captured in the intradomicile and in the peridomicilio (Fig 5).

Collections with PHBC resulted more effective with respect to the number of adult mosquitoes collected (n = 1,024, 96.3%), in comparison to the number of mosquitoes collected with LTs (n = 39, 3.7%). The majority of mosquitoes were collected during May, when the rainy season begins, and October (n = 173, 16.3%) and November (n = 208, 19.6%); which traditionally are the rainiest months of the year (Table 4). Collections during the dry season months registered a lower number of captured adult mosquitoes, March (n = 47, 4.4%) and April (n = 18, 1.7%). The studied communities that registered the major percentage of captured mosquitoes were Bajo Chiquito (n = 589, 55.4%) and Marraganti (n = 345, 32.5%), followed by Pueblo Tortuga (n = 64, 6.0%) and Alto Playona (n = 52, 4.9%). In turn, the localities in which a larger number of mosquito species was collected were Bajo Chiquito with seven species, followed by Marraganti and Alto Playona with five species, respectively.

**Table 4:** Number of *Anopheles* mosquito species captured with protective human-baited capture and CDC light traps per month in the communities under study in the Darien Region, Panama, 2016.

Species mosquito	March	April		May		June	July	August	September	October	November	Total
*An*. *(Nys*.*) darlingi*	7	8		105		35	32	41	41	103	106	**478**
*An*. *(Nys*.*) albimanus*	30	7		80		47	40	35	42	70	102	**453**
*An*. *(Ano*.*) pseudopunctipennis s*.*l*.	10	3		58			7	1	8			**87**
*An*. *(Ano*.*) punctimacula s*.*l*.				3								**3**
*An*. *(Nys*.*) oswaldoi s*.*l*.				7				8				**15**
*An*. *(An*.*) apicimacula*				1								**1**
*An*. *(An*.*) malefactor*									1			**1**
*An*. *(Nys*.*) triannulatus s*.*l*.				2					1			**3**
*An*. *(Nys*.*) strodei*				1								**1**
*Anopheles (Nys*.*) spp*.						5			5			**10**
*Anopheles (Ano*.*) spp*.				1								**1**
*Anopheles spp*.						10						**10**
**Total mosquitoes per month**	**47**		**18**		**258**	**97**	**79**	**85**	**98**	**173**	**208**	**1063**

### Detection and genotype of natural *Plasmodium spp*. infection in *Anopheles spp*.

A total of 574 the 1,063 adult females collected were selected and processed according to the quantity, mosquito species, collecting date, capture type (PHBC or LTs) and study site for the detection and genotype of *Plasmodium* natural infections by PCR. The samples were grouped in 148 anophelines pools per species of varying size (between 1 and 5 individuals). The contribution of all the species selected for this test and number of the pools analyzed were as follow: *An*. *(Nys*.*) albimanus* (n = 179, 42 pools), *An*. *(Nys*.*) darlingi* (n = 305, 68 pools), *An*. *(Ano*.*) punctimacula s*.*l*. (n = 29, 12 pools), *An*. *(Ano*.*) pseudopunctipennis s*.*l*. (n = 43, 15 pools), *An*. *(Nys*.*) oswaldoi s*.*l*. (n = 9, 3 pools), *An*. *(Ano*.*) malefactor* (n = 1, 1 pool), *Anopheles (Nys*.*) spp*. (n = 7, 6 pool) and *Anopheles (Ano*.*) spp*. (n = 1, 1 pool).

From 148 pools tested, 20 (13.5%) resulted positive for *P*. *vivax* by the ssrRNA PCR technique. These *P*. *vivax* positive pools were comprised of nine positive pools from *An*. *(Nys*.*) albimanus* (6.1%) and 11 positive pools from *An*. *(Nys*.*) darlingi* (7.4%). The overall *Plasmodium* prevalence was 0.037 (95% Cl, 0.0232–0.0552); 0.0551 (95% Cl, 0.0269–0.0974) in *An*. *(Nys*.*) albimanus* and 0.0384 (95% Cl, 0.0201–0.0649) in *An*. *(Nys*.*) darlingi* (Table 5).

**Table 5:** *Plasmodium vivax* prevalence and positive pool numbers from *An*. *(Nys*.*) albimanus* and *An*. *(Nys*.*) darlingi* mosquitoes collected in Darien, Panama.

Species	Prevalence (95% CL)	Pools (+/n)	Abundance (N)
*An*. *(Nys*.*) albimanus*	0.0551 (0.0269–0.0974)	9/42	179
*An*. *(Nys*.*) darlingi*	0.0384 (0.0201–0.0649)	11/68	305
Total	0.037 (0.0232–0.0552)	20/148	574

The geographical distribution of the positive An. *(Nys*.*) albimanus* pools was: one *P*. *vivax* pool in the community of Pueblo Tortuga (0.6%), three positive pools in Marraganti (1.7%), two pools in Bajo Chiquito (1.1%) and three pools in Alto Playona (1.7%). As for the 11 *P*. *vivax*-positive *An*. *(Nys*.*) darlingi* samples, seven positive pools were from the community of Bajo Chiquito (4.0%), two pools from Marraganti (1.1%) and two pools from Alto Playona (1.1%) (Fig 1). All other species were negative for *P*. *vivax* by the ssrRNA PCR technique. All collected samples tested negative for *P*. *falciparum*. Most positive samples were captured in the peridomicile, and only two samples from *An*. *(Nys*.*) albimanus* were collected in the extra-domicile using LTs in the community of Alto Playona.

**Fig 6:**
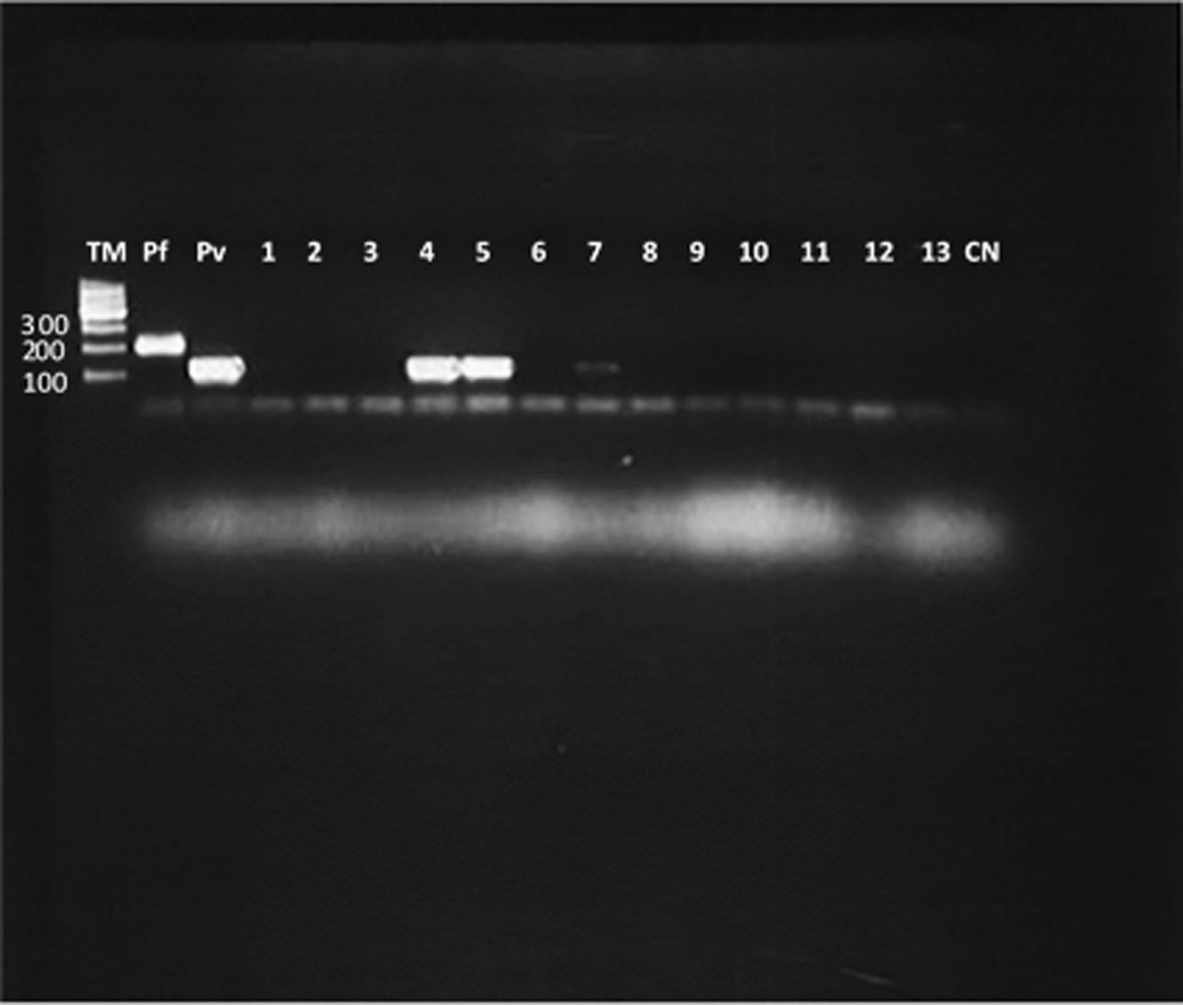
Nested PCR agarose gel electrophoresis to detect infection by *Plasmodium spp*. in pool of *Anopheles spp*. TM: DNA ladder (100bp, Qiagen), Pf: *P*. *falciparum* positive control (205 bp), Pv: *P*. *vivax* positive control (120 pb), Lane 1,3,6,8,9: pool samples of *An*. *(Nys*.*) albimanus* negative, lane 2,10,11,12,13: pool sample of *An*. *(Nys*.*) darlingi* negative, lane 4 and 5: pool sample *An*. *(Nys*.*) darlingi* positive for *P*. *vivax*, lane 7: pool sample *An*. *(Nys*.*) albimanus* positive for *P*. *vivax*, CN: negative control of PCR.

**Fig 7:**
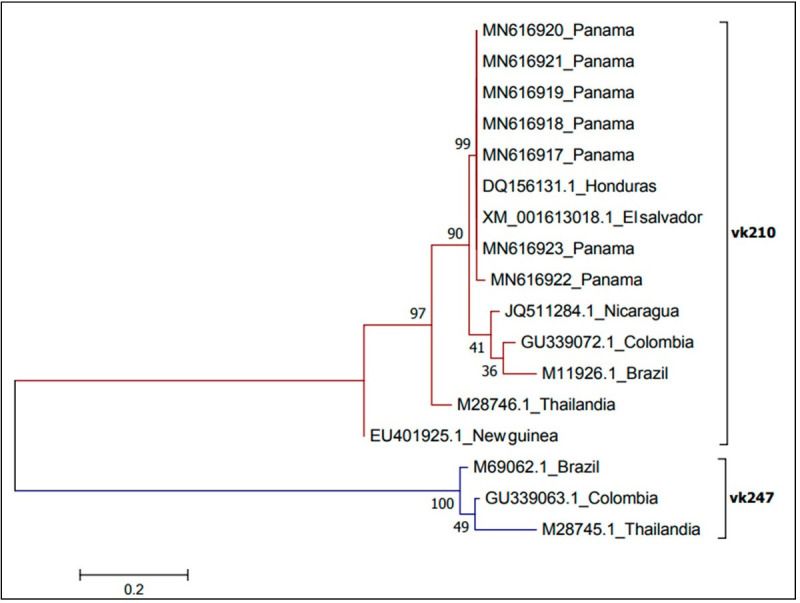
Phylogenetic analysis of *P*. *vivax* CSP gene. The phylogeny tree was constructed with the neighbor-joining method with the JTT (John Taylor Torton) model using the MEGA 3 program.

From the positive pool samples, it was possible to genotype *P*. *vivax* in seven of them, four from *An*. *(Nys*.*) darlingi* and three from *An*. *(Nys*.*) albimanus* by sequencing the csp gene marker. Amplified products were in the range between 700 to 800 bp and their sequence analysis determined that they were homologous to the VK210 variant, grouping together with other isolates from Central America (Fig 6). Sequences from this study are available at the GenBank database under the accession numbers: MN66917, MN66918, MN66919, MN66920, MN66921, MN66922, MN66923 (Fig 7).

In addition, analysis of *Anopheles* COI gene confirmed the identity of *An*. *(Nys*.*) darlingi* and *An*. *(Nys*.*) albimanus* as the species naturally infected with *P*. *vivax* in this study. These sequences were deposited in the GenBank database under the accession numbers: MT706455 for *An*. *(Nys*.*) darlingi* and MT706456 for *An*. *(Nys*.*) albimanus*.

## Discussion

### Biting activity

The diversity of *Anopheles* mosquitoes in Panama is favored by a vast variety of habitats and environmental conditions that support the development, dispersion and persistence of these mosquito populations. The abundance and human feeding preference are among the main characteristics that define a mosquito species as an effective mosquito vector [47]. Nevertheless, the composition and frequency of mosquito species vary between sampling sites and seasons, making it even more important the need for a constant monitoring to better understand these parameters [47, 48]. Our data shows that *An*. *(Nys*.*) albi*manus was the dominant species in four out of the five sampled sites. This species is described as predominantly exophagic, with preference for animals and with hematophagy occurring during the entire night [48].

In turn, *An*. *(Nys*.*) darlingi* was the species that registered the major number of captured mosquitoes and was dominant in one of the collection sites (Bajo Chiquito). *Anopheles (Nys*.*) darlingi* is considered one of the most effective primary vectors due to its high anthropophilic behavior, high abundance in certain areas, susceptibility to infection by several *Plasmodium* species and plastic behavior [49]. This species can also adapt to diverse habitats, including habitats developed because of human activities [50, 51]. Our study showed that both mosquito species (*An*. *(Nys*.*) darlingi* and *An*. *(Nys*.*) albimanus*) had the greatest density and biting activity between 18:30 and 19:30 hours, diminishing towards midnight as described in previous studies [22, 28, 51]. A study in Colombia also observed that the greatest intradomicile and peridomicile biting activity of *An*. *(Nys*.*) darlingi* was between 18:00 and 19:00 hours [24, 52], similarly to our data and those described in French Guyana and Brazil [46]. Furthermore, studies conducted in Barú municipality, a locality in Panama bordering Costa Rica, demonstrated that *An*. *(Nys*.*) albimanus* had greater abundance and biting frequency than *An*. *(Ano*.*) punctimacula s*.*l*. between 18:30 and 19:30 hours [29]. Overall, our entomological data from the intradomicile and peridomicile collections suggest that there is higher transmission risk between 18:00 and 22:00 hours, when the anophelines population is higher. Therefore, considering the abundance and high *Plasmodium* natural infection of these two species in most collecting sites, our data is highly suggestive that *An*. *(Nys*.*) darlingi* and *An*. *(Nys*.*) albimanus* are acting as the main malaria vectors in this area. Nevertheless, other malaria vector species found in this study and also known to be present in other neotropical countries, such as *An*. *(Ano*.*) pseudopunctipennis s*.*l*., *An (Nys*.*) oswaldoi s*.*l*., *An*. *(Ano*.*) punctimacula s*.*l*. and *An*. *(Ano*.*) triannulatus s*.*l*. [28, 53], could also be implicated in the local transmission.

The intra and peridomicile PHBC rates for both *An*. *(Nys*.*) darlingi* and *An*. *(Nys*.*) albimanus* were similar, without significant differences in the biting activity percentages, confirming the exophagic and endophagic behavior of these species [54]. While the PHBC for both species was respectively 0.20 to 0.50 bites per night, other studies have found more variable biting rates for *An*. *(Nys*.*) darlingi* [55, 56]. The low density recorded in these two species may be due to variations in temperature, rainfall, human activity, proximity and availability of breeding sites and other environmental factors [57, 58]. It could also be due to the collection period during the study, thus it was not possible to establish if these populations present a greater activity at other hours of the night, as it has been described in other populations of the region [24]. For instance, rates of 0.1–15.1 BHN [22] and 2.2–55.5 BHN [54] were observed in Colombia and 53.8 to 837.7 BHN in the Brazilian amazon [59]. Also, studies conducted with *An*. *(Nys*.*) albimanus* in eight communities during a malaria outbreak in Panama displayed a PHBC of 1.9 to 30.9 BHN [29]. A previous study conducted in 31 endemic sites in Panama found that the BHN oscillated between 2.4 and 10.2 BHN [8]. Further studies conducted in the endemic regions of the Colombian Pacific found that *An*. *(Nys*.*) albimanus* had the highest biting activity between 17:00 and 19:00 hours, reaching a PHBC of 38.4 bites/night [60]. In general, our results indicated a lower BHN rate compared to reports conducted in other Neotropical countries. It is thus necessary to conduct prolonged studies to estimate the densities and understand the biting behavior of this mosquito species in the different Panamanian regions.

Our monthly mosquito collections indicated that the greatest anophelines density was obtained in the months of May, October and November (rainy season), with predominance of *An*. *(Nys*.*) darlingi* and *An*. *(Nys*.*) albimanus*. The lowest densities for both species were observed in March (dry season). Several studies have described patterns of seasonal fluctuation in the abundance and frequency of anophelines vectors, showing greater densities during the rainy months and lower densities during the dry months [61, 62]. Few studies have demonstrated no correlation between the levels of precipitation and the density of anophelines mosquitoes [41, 58]. On the other hand, it has also been reported that an increase in density can be observed on the transition periods between the dry and rainy seasons [63, 64]. However, it has been proposed that the changes in vector density not only depends on the aforementioned factors but rather it responds to an interaction between the availability of breeding sites, the levels of water sources and other environmental variables [48].

In this study, captures with PHBC were more effective compared to LTs. In general, catches with LTs outdoors are less efficient compared to PHBC. They are used as a supplement to captures with PHBC, with the purpose of having a greater opportunity and probability of capturing a larger number of anophelines species. For instance, in a study carried out in Córdoba, Colombia, only six species of *Anopheles* were captured intradomicile and in the peridomicile with LTs. This number of captured species was small considering that there are 20 species recorded in that region out of a total of 47 species registered in Colombia. In addition, trap catches require that the traps do not harm captured mosquitoes [54, 65]. Therefore, captures with PHBC have become the most widely used in malaria studies [43, 66, 67].

### Detection of natural *Plasmodium* infection in *Anopheles* spp.

The communities that yielded *P*. *vivax* positive mosquito pools also registered active malaria transmission during our study, with the highest number of cases observed during the transition period between the rainy season and the dry season [5]. The dynamics of malaria epidemics are strongly influenced by climate. In particular, at the geographical limits of its distribution, malaria transmission is driven by environmental factors changes as temperature, rain and humidity [68].

Interestingly, our study allowed us to detect for the first time in the country the natural *P*. *vivax* infection of *An*. *(Nys*.*) darlingi*, and to suggest its potential incrimination as a malaria vector in this region of Panama. At the same time, our results showed that two other species historically considered to be important malaria vectors, *An*. *(Ano*.*) punctimacula s*.*l*. and *An*. *(Ano*.*) pseudopunctipennis s*.*l*., were not infected *Plasmodium*. Thus, their epidemiological relevance as vectors could not be confirmed for this region. This could have been due to the low number of individuals of both species captured during this study.

The genus *Anopheles* is present around the world with about 465 species, of which 41 species are important malaria vectors [69]. Although different mosquito species can be involved in malaria transmission in different regions [58, 70, 71], little is known about the contribution of each species in malaria prevalence in heterogenic environments. This is especially true given that each species has unique developmental, ecological and behavioral characteristics. A study conducted in Brazil indicated that this scenario becomes more complex with the presence of three different malaria parasite species [72, 73] in the same geographical region. *Anopheles (Nys*.*) albimanus* and *An*. *(Ano*.*) punctimacula s*.*l*. are considered as the primary and secondary malaria vectors, respectively in Panama. *Anopheles (Nys*.*) albimanus* is the most prevalent in the endemic regions [8]. A recent study reported the detection, via PCR, of natural *P*. *vivax* infections in *An*. *(Nys*.*) albimanus* mosquitoes collected from the communities of Achutupo and Playon Chico, both in the indigenous comarca of Guna Yala [5]. In addition, other studies have detected *P*. *vivax* natural infections in *An*. *(Nys*.*) albimanus* (via ELISA tests) and in *An*. *(Ano*.*) punctimacula s*. *l*. (through PCR assays) collected in Bocas del Toro province [74]. In another study *An*. *(Nys*.*) albimanus* mosquitoes was found naturally infected with *P*. *vivax* in samples collected in the community of Ipeti Guna, located in the Madungandi comarca [44].

The determination of natural *Plasmodium* infection of anophelines mosquitoes is an important component in the assessment of different mosquito species as malaria vectors [11]. Nevertheless, there are other anophelines species that could be important malaria vectors in the different endemic regions of Panama. Previous studies conducted in Panama have detected natural infection *P*. *vivax* or *P*. *falciparum* in *An*. *(Nys*.*) albimanus*, *An*. *(Nys*.*) argyritarsis*, *An*. *bachmanni* [*syn*. *An*. *(Nys*.*) triannulatus*] and *An*. *(Ano*.*) punctimacula s*.*l*. [syn. *An*. *(Ano*.*) malefactor*], while the following mosquitoes were experimentally infected: *An*. *(Ano*.*) pseudopunctipennis s*.*l*., *An*. *(Nys*.*) tarsimaculatus* [*Syn*. *An*. *(Ano*.*) aquasalis*], *An*. *(Ano*.*) apicimacula*, *An*. *(Ano*.*) eiseni* and *An*. *(Ano*.*) neomaculipalpus* [12–16].

Another important result is the distribution of *An*. *(Nys*.*) darlingi* in new areas and communities far from Jaque and Biroquera, where it was first reported in the country [20]. The new locations are situated further into the Darién southeast region and closer to the Colombian border. These new records extend the distribution of *An*. *(Nys*.*) darlingi* in Panama. The detection of natural *An*. *(Nys*.*) darlingi* infection, increases its population spread to three communities of the five evaluated. In addition, it suggests its participation in the active *P*. *vivax* malaria transmission, in a similar fashion than *An*. *(Nys*.*) albimanus* in this region of Panama.

Darién, as a Panamanian region bordering with Colombia, shares part of its entomofauna, including anophelines malaria vectors. Epidemiological and entomological studies conducted in Colombia have incriminated 12 anophelines species as malaria vectors. Three of these species are primary malaria vectors: *An*. *(Nys*.*) albimanus*, *An*. *(Nys*.*) darlingi* and *An*. *(Nys*.*) nuneztovari s*.*l*. [75]. The other nine species are secondary or local vectors: *An*. *(Ano*.*) pseudopunctipennis s*.*l*., *An*. *(Ano*.*) punctimacula s*.*l*., *An*. *(Ano*.*) calderoni*, *An*. *(Ano*.*) neomaculipalpus*, *An*. *(Ker*.*) pholidotus*, *An*. *(Ker*.*) neivai s*.*l*., *An*. *(Nys*.*) rangeli*, *An*. *(Nys*.*) benarrochi*, and *An*. *(Nys*.*) oswaldoi s*.*l*. [75, 76].

### *Plasmodium spp*. genotyping

Genetic studies of circulating malaria parasite populations in humans and vectors reveal critical information about the epidemiology and dynamics of disease transmission and offer tools to support control and elimination efforts [77]. In the present study, we only identified the *P*. *vivax* allelic variant VK210 naturally infecting *An*. *(Nys*.*) albimanus* and *An*. *(Nys*.*) darlingi* mosquitoes circulating in the studied areas. It is possible, however, the lack of detection of the VK247 variant might be due the low number of samples analyzed, as this variant has been described circulating in malaria endemic communities near the border with Colombia close to the ones studied [78].

There are studies related to the diversity in infectivity of allelic variants and the susceptibility of mosquito species to different allelic variants, which may explain the detection patterns at the species level, where *An*. *(Nys*.*) albimanus* seems to be susceptible to VK210 and VK247 infection [52]. However, other studies conducted in anophelines mosquitoes from Brazilian Amazon, observed the distribution of *P*. *vivax* VK247 changed over time in the main malaria vectors on the Brazilian Amazon. *Anopheles (Nys*.*) darlingi* was abundant in certain localities while *An*. *(Nys*.*) albitarsis s*.*l*. in anothers, which highlights the importance of entomological studies for the control of human malaria [79]. Investigations carried out in Mexico considered that *An*. *(Nys*.*) albimanus* is more susceptible to VK210 infection and *An*. *(Ano*.*) pseudopunctipennis* was more susceptible to VK47 [80].

Studies on genotyping parasite populations have the power to reveal key information about the epidemiology and dynamics of malaria transmission, with the potential to offer tools to support control and elimination efforts [81]. In future studies, is also critical to evaluate the epidemiologic role on malaria transmission of other anophelines species previously described in the Darien region such as *An*. *(Nys*.*) triannulattus s*.*l*., *An*. *(Nys*.*) oswaldoi s*.*l*., *An*. *(Ano*.*) pseudopunctipennis s*.*l*., *An*. *(Ano*.*) punctimacula s*.*l*., and *An*. *(Ano*.*) malefactor*. Additional molecular studies of malaria allelic variants circulating in Panama at the local and regional are necessary to expand our current knowledge, determine parameters that affect malaria transmission dynamics, and to develop novel malaria control strategies [82, 83]. Previously in *An*. *(Nys*.*) albimanus* collected from 2006–2007 in Bocas del Toro, Panama, nine pools were detected naturally infected with *P*. *vivax* by an ELISA test (three pools with the VK210 variant and six with the VK247 variant) [74].

An important limitation of this study was that it was not possible to carry out salivary gland dissections for the detection of *P*. *vivax* sporozoites in *An*. *(Nys*.*) albimanus* and *An*. *(Nys*.*) darlingi*. Therefore, we were not able to corroborate and confirm the PCR results that suggested the incrimination of *An*. *(Nys*.*) albimanus* and *An*. *(Nys*.*) darlingi* in malaria transmission at the local level. However, it should be noted that *An*. *(Nys*.*) Darlingi* has been sufficiently proved for its great vector capacity through various studies, classifying it as the most efficient malaria-transmitting species in the Americas.

In conclusion, this study provides important new information on the transmission dynamics associated with mosquito vectors in the Darien region. The data show that the most abundant and distributed species were *An*. *(Nys*.*) albimanus* and *An*. *(Nys*.*) darlingi*, which were also found coexisting in the same geographical area. Furthermore, this study reports for the first time the detection of natural *P*. *vivax* infection in *An*. *(Nys*.*) darlingi*, its incrimination in malaria transmission and its identification in new areas of Darien. This study also detected the *P*. *vivax* variant VK210 in *An*. *(Nys*.*) albimanus* and *An*. *(Nys*.*) darlingi* mosquitoes. Nevertheless, it is necessary to evaluate the role of other anophelines species, such as *An*. *(Nys*.*) triannulattus s*.*l*., *An*. *(Nys*.*) oswaldoi s*.*l*., *An*. *(Ano*.*) pseudopunctipennis s*.*l*., *An*. *(Ano*.*) punctimacula s*.*l*. and *An*. *(Ano*.*) malefactor*, in malaria transmission dynamics given their epidemiological importance has yet to be determined. In addition, results of this research add important new entomological information that should be considered in transmission dynamic studies, and in surveillance/control strategies in the Darien region. Specifically, at the local level, these findings provide a new geographical range for mosquitoes, some of which could be acting as local vectors. Our findings highlight the need for additional studies to expand our knowledge on the behavior, spatial/temporal distribution, and malaria transmission dynamics by *An*. *(Nys*.*) darlingi* in this Panamanian region.

## Supporting information

S1 Data (XLS)

## List of abbreviations

MINSA: Ministry of Health
NMP: National Malaria Program
ED: Epidemiology Department
ICGES: Instituto Conmemorativo Gorgas de Estudios de la Salud
MEF: Ministry of Economy and Finance of Panama
WHO: World Health Organization
PAHO: Pan American Health Organization
CDC: Centers for Disease Control and Prevention
PHBC: Protective human-baited capture
IR: Mosquito infection rate
PCR: Polymerase Chain Reaction
ELISA: Enzyme-linked immunosorbent assay
DNA: Deoxyribonucleic acid
RNA: Ribonucleic acid
CEP: Circumsporozoite protein
